# Social justice in medical education: a student-led approach to addressing COVID-19 vaccine equity in the Hispanic/Latinx community

**DOI:** 10.1080/10872981.2023.2241169

**Published:** 2023-07-27

**Authors:** Audrey V. Adler, Haley R. Nadone, Mirabel E. Dafinone, Kevin C. Facemyer

**Affiliations:** University of Nevada, Reno School of Medicine, Reno, Nevada, USA

**Keywords:** social justice, health equity, health disparities, diversity equity and inclusion, COVID-19

## Abstract

The current healthcare system disproportionately affects vulnerable populations, leading to disparities in health outcomes. As a result, medical schools need to equip future physicians with the tools to identify and address healthcare disparities. The University of Nevada, Reno School of Medicine implemented a Scholarly Concentration in Medical Social Justice (SCiMSJ) program to address this issue. Three medical students joined the program and pioneered a project to address the equitable vaccine distribution within the local Hispanic/Latinx community. After identifying the disparity in vaccine uptake and high levels of vaccine hesitancy, they collaborated with local organizations to address vaccine misinformation and accessibility. They organized outreach events, provided vaccine education, and hosted a vaccine clinic at a Catholic church with a high Hispanic/Latinx congregation. Through their efforts, they administered 1,456 vaccines. The estimated economic and societal impacts of their work was 879 COVID-19 cases avoided, 5 deaths avoided, 45 life years saved, and $29,286 in economic value. The project’s success highlights the effectiveness of a student-led approach to promote skill development in social justice training. Leadership skills and coalition building were crucial in overcoming resource limitations and connecting organizations with the necessary volunteer force. Building trust with the Hispanic/Latinx community through outreach efforts and addressing vaccine hesitancy contributed to the well-attended vaccine clinic. The project’s framework and approach can be adopted by other medical students and organizations to address health disparities and improve health outcomes in their communities.

## Educational problem

Patients of color, low-income patients, women, and other vulnerable populations suffer and die disproportionately due to the shortcomings of our current healthcare system [1,2]. The COVID-19 pandemic unmasked some of these deeply rooted disparities. Now more than ever, rising physicians need to be aware of healthcare disparities and be equipped with the cultural humility to care for vulnerable patients [3]. Empowering students to enter the workforce by fostering their awareness of the social determinants of health, and developing their skills in addressing healthcare disparities through public health intervention, is a crucial mission of today’s medical schools [2,4,5].

## Innovation

Early in the COVID-19 pandemic, the University of Nevada, Reno School of Medicine piloted and implemented a Scholarly Concentration in Medical Social Justice (SCiMSJ) to provide medical students training in addressing health disparities. The objective for year 1 was to identify and address a local health disparity exacerbated by the pandemic through a student-led approach. As three medical students, we joined the program under the supervision of a faculty mentor.

## Methods

First, we investigated local health disparities through data analysis and research. We proposed potential interventions, estimated their health and economic impact, and received feedback from our mentor and community faculty. After identifying disparities within the local Hispanic/Latinx community in terms of COVID-19 burden [6–10] and vaccine uptake rates [11–14], we decided on a project goal of addressing the equitable vaccine distribution within the local Hispanic/Latinx community.

Next, we planned a culturally sensitive intervention. To overcome limitations in time, resources, and scope of practice we sought support and collaboration from other local groups. We formed a partnership with #COVIDCrew, a nonprofit organization from the Trudy Larson MD Institute for Health Impact and Equity. Together our groups investigated factors contributing to the lagging vaccination rates and explored targeted interventions to address misinformation, provide education, and meet people where they were most comfortable for effective vaccine distribution. We identified a Catholic church with a high Hispanic/Latinx congregation and hosted six recurring outreach events to answer vaccine-related questions before and after service. We educated a task force of bilingual medical students to help with translation. Second, we provided vaccine education and offered translational services at two separate vaccine clinics hosted by Immunize Nevada and the City of Reno targeting the Hispanic/Latinx community. Lastly, we planned a 2-day vaccine clinic at the same Catholic church to improve accessibility.

## Intervention outcome

We assessed our project’s effectiveness by estimating the health and economic impacts according to the number of vaccines we helped administer (1,456 vaccines). We identified a study which estimated the impact of COVID-19 vaccines over 3.5 years in the United States [14]and applied ratios of outcomes per number vaccinated to the number vaccinated through our work [15]. Our project’s estimated impact is 879 COVID-19 cases avoided, 5 deaths avoided, 45 life years saved, and $29,286 in economic value from avoiding COVID-19 infections and resuming social and economic activity more quickly.

## Conclusion

There were three important lessons from the SCiMSJ curriculum structure and our specific project that promoted success in terms of social justice training and addressing local health disparities via public health initiative.

First, a key component was the student-led approach because this promoted student empowerment and skill development in social justice training. This is supported by other medical schools which have shown student-led social justice programs to enable medical students to examine the concepts of social medicine and engage in meaningful problem solving [11,12]. Our project’s quantifiable health and economic impacts support the notion that students can effectively recognize health inequities and employ innovative public health interventions that result in meaningful change.

Second, specific to our project, we discovered the power of leadership skills and coalition building to connect resource-limited organizations with the necessary volunteer-force to proactively address healthcare disparities. Our group was initially limited by size and lack of resources; meanwhile public and community health organizations were limited by workforce shortages and lack of Spanish-speakers. Therefore, we overcame these challenges and increased our project’s impact by coordinating medical students as translators and volunteers to support existing organizations.

Third, another factor critical to our project’s success was the trust built with the Hispanic/LatinX community, evident by the large turnout at our 2-day vaccine clinic (673 individuals). Because we had identified COVID-19 vaccine hesitancy in this community [11–14], we explored targeted interventions to meet people where they were at to provide education and answer questions related to the vaccine before bringing in the vaccine itself. We identified a Catholic church with a high Hispanic/LatinX congregation as a beacon of trust in the community and decided to first build a relationship through recurring outreach events. We educated our task force of medical students fluent in Spanish on motivational interviewing techniques to address vaccine hesitancy given research supporting its effectiveness [16,17]. Removing the language barrier was critical to discuss the COVID-19 vaccines and addressing misinformation through an empathetic, patient-centered approach.

The trust developed through these outreach efforts contributed to our well-attended vaccine clinic. In return, we fostered a sense of cultural humility by listening to concerns and questions about the COVID-19 vaccine. These factors highlight the importance of meeting populations where they feel most comfortable, speaking their language and addressing misinformation and hesitancy prior to offering a vaccine clinic.

In summary, our project’s success supports the effectiveness of a student-led approach in medical social justice training. Key takeaways include the power of leadership skills, coalition building, and cultural humility. These lessons, in addition to our project’s framework and approach, are applicable to other schools and learners interested in pioneering their own public health initiatives. Specifically, the framework involved challenging students to address a local health disparity and the approach consisted of preparatory investigation, estimation of impact, community collaboration, and action. Combined, this process can be adopted by other medical students, clinicians, and organizations looking to offer training opportunities in medical social justice to improve health outcomes in their own communities.

## Previous presentations

1. Adler AV, Nadone HR, Dafinone ME, Facemyer KC. Co-authors Sande DV, Dupin ZD. *Overcoming Barriers To COVID-19 Vaccine in Hispanic/Latinx Community*. Nevada Academy of Family Physicians Winter CME Meeting Poster Competition. Stateline, NV, 2022.
2. Adler AV, Nadone HR, DafinoneME, Facemyer KC. Co-authors Sande DV, Dupin ZD. *Medical Social Justice:Overcoming Barriers to the COVID-19 Vaccine in The Hispanic/ Latinx Community*. Poster presented at the 2022 Innovations in Medical Education Online Conference.Hosted by Keck School of Medicine of USC. Virtual, 2022.
3. Adler AV, Nadone HR, DafinoneME, Facemyer KC. Co-authors Sande DV, Dupin ZD. *Social Justice in MedicalEducation: A Student-Led Approach to Addressing COVID-19 Vaccine Hesitancy inThe Hispanic/ Latinx Community*. Poster presented at the 2022 Western Groupon Education Affairs (WGEA) Regional Meeting. Portland, OR, 2022.
4. Adler AV, Nadone HR, Dafinone ME, Facemyer KC. Co-authors Sande DV, Dupin ZD. *Social Justice in Medical Education: A Student-Led Approach to Addressing COVID-19 Vaccine Hesitancy in The Hispanic/ Latinx Community*. Poster presented at the 2022 Nevada State Medical Association Annual Meeting. Las Vegas, NV, 2022.
5. Adler AV, Nadone HR, Dafinone ME, Facemyer KC. Co-authors Sande DV, Dupin ZD. *Social Justice in Medical Education: A Student-Led Approach to Addressing COVID-19 Vaccine Hesitancy in the Hispanic/ Latinx Community*. Poster presented at the Learn Serve Lead 2022: The AAMC Annual Meeting Conference. Nashville, TN, 2022.
